# Macroalgal Extracts Induce Bacterial Assemblage Shifts and Sublethal Tissue Stress in Caribbean Corals

**DOI:** 10.1371/journal.pone.0044859

**Published:** 2012-09-13

**Authors:** Kathleen M. Morrow, Raphael Ritson-Williams, Cliff Ross, Mark R. Liles, Valerie J. Paul

**Affiliations:** 1 Auburn University, Department of Biological Sciences, Auburn University, Auburn, Alabama, United States of America; 2 Smithsonian Marine Station, Smithsonian Institution, Fort Pierce, Florida, United States of America; 3 Department of Biology, University of North Florida, Jacksonville, Florida, United States of America; Dowling College, United States of America

## Abstract

Benthic macroalgae can be abundant on present-day coral reefs, especially where rates of herbivory are low and/or dissolved nutrients are high. This study investigated the impact of macroalgal extracts on both coral-associated bacterial assemblages and sublethal stress response of corals. Crude extracts and live algal thalli from common Caribbean macroalgae were applied onto the surface of *Montastraea faveolata* and *Porites astreoides* corals on reefs in both Florida and Belize. Denaturing gradient gel electrophoresis (DGGE) of 16S rRNA gene amplicons was used to examine changes in the surface mucus layer (SML) bacteria in both coral species. Some of the extracts and live algae induced detectable shifts in coral-associated bacterial assemblages. However, one aqueous extract caused the bacterial assemblages to shift to an entirely new state (*Lobophora variegata*), whereas other organic extracts had little to no impact (e.g. *Dictyota* sp.). Macroalgal extracts more frequently induced sublethal stress responses in *M. faveolata* than in *P. astreoides* corals, suggesting that cellular integrity can be negatively impacted in selected corals when comparing co-occurring species. As modern reefs experience phase-shifts to a higher abundance of macroalgae with potent chemical defenses, these macroalgae are likely impacting the composition of microbial assemblages associated with corals and affecting overall reef health in unpredicted and unprecedented ways.

## Introduction

Reef-building corals harbor diverse eukaryotic and prokaryotic microorganisms that form dynamic mutualistic, parasitic, and commensal associations with the coral host [1]–[6]. Although many of the roles that microorganisms play in coral physiology and immune function remain unknown, recent evidence suggests that corals harbor specific and beneficial microbial assemblages [1], [3], [5], [7]. Some microbes can provide protection against bacterial or fungal infection [8] by the production of antibiotics or by filling a niche that otherwise would be open to infection by opportunistic pathogens [9]. Detecting when and why shifts occur in healthy coral-microbial assemblages is to a large extent unknown. If the factors that impact coral-microbial associations are better defined this might enable a better understanding of the roles that these microorganisms play in maintaining coral health and whether specific microorganisms could serve as an indicator of negative environmental changes.

Macroalgae are becoming increasingly abundant competitors of corals (reviewed in [10], [11]), and as coral-algal interactions increase, there is greater potential for macroalgae to mediate changes in the coral-associated microorganisms and cause significant coral stress. Macroalgae utilize both physical and biochemical mechanisms to compete with corals and other reef invertebrates (reviewed in [10], [12]). Allelopathy has been specifically implicated in the reduction of coral larval metamorphosis and settlement [13], [14], as well as bleaching, decreased photosynthesis, and occasional mortality of adult corals [15]–[17]. Cellular diagnostics has only recently been applied to coral tissues, but provides a systematic approach to examining the functionality of cellular biomarkers. Shifts in cellular conditions are shown to reflect overall cellular integrity and performance [18], [19]. While several studies have examined the sublethal stress response of adult corals exposed to anthropogenically-based pollutants [19]–[21] as well as natural global stressors [22], the sublethal stress response of adult corals exposed to macroalgal extracts has not been assessed until now.

Coral reefs dominated by macroalgae experience lower levels of oxygen and higher abundances of heterotrophic bacteria, including potential pathogens, within the overlying seawater [23], [24]. Organic carbon exuded by macroalgae naturally stimulates microbial colonization and growth near algal surfaces and within the water column [25], [26]. Excess labile carbon in the water column is believed to stimulate microbial activity [23], and is correlated with zones of hypoxia and tissue mortality at coral-algal interfaces [27], [28]. Macroalgae can be challenged by microbial fouling and disease that sometimes causes mass mortalities [29]. In contrast, macroalgae can also host diverse and specific assemblages of beneficial microorganisms [30], that can utilize algal-derived sugars and assist in the decay process of algal fronds. These observations coupled with recent antibacterial assays revealed that macroalgae often mediate harmful microorganisms through the production of surface-associated chemical defenses [31]–[34]. Actively responding to microbes, macroalgae release reactive oxygen species (ROS), and produce defensive secondary metabolites that prevent bacterial communication, swarming, and attachment to surfaces [26], [35]–[37]. Recently, antifungal chemical defenses including bromophycolides and callophycoic acids were isolated from the red macroalga *Callophycus serratus* [38]. Another red macroalga, *Bonnemaisonia hamifera,* can mediate epibiotic bacteria by producing broad-spectrum growth inhibiting secondary metabolites [39]. Extracts from Caribbean macroalgae both stimulated and inhibited coral reef-associated bacterial cultures [40], suggesting that macroalgae and the compounds they produce can alter microbial assemblages associated with reef-building corals. Numerous studies demonstrate *in vitro* that macroalgae are a rich source of antifouling and antibacterial compounds (reviewed in [41]), but few studies have examined the *in situ* role of allelopathy in interactions between algae, macroinvertebrates and their associated microorganisms.

The objective of this study was to determine whether macroalgal extracts caused a detectable stress response in coral tissues and/or a shift in coral-associated bacterial assemblages. We applied crude extracts from three common Caribbean macroalgae directly to *Montastraea faveolata* and *Porites astreoides* corals on reefs in both Florida and Belize. Whole macroalgal thalli were also applied to coral tissues for comparison with extract-exposed tissues. Cellular diagnostics were used to examine whether macroalgal compounds caused sublethal stress to coral tissues. Denaturing gradient gel electrophoresis (DGGE) was used to examine changes in the bacterial assemblages associated with the surface mucus layer (SML) of both coral species. Macroalgal extracts caused sublethal stress in several treatments and a detectable shift in the bacterial assemblages in 44% of coral-extract interaction pairs. An aqueous extract from the brown macroalga *Lobophora variegata* caused coral-bacterial assemblages to shift to an entirely new composition and caused an increase in detoxification enzymes within both coral species.

## Methods

### Macroalgal Collection and Extraction

Macroalgae were collected by hand on SCUBA at depths of 8–15 m from 2006–2008 in Belize, the Florida Keys, and St. Thomas, U.S. Virgin Islands (Table 1). Macroalgal extracts from samples collected in the Florida Keys (*H. tuna*) and St. Thomas (*L.* variegata) were applied to corals in Florida (Table 1). Extracts from samples collected in Belize (*Dictyota* sp., *H. tuna*) and St. Thomas (*L.* variegata) were applied to corals in Belize (Table 1). All three macroalgal species are abundant on Florida and Belize reefs. All samples were placed in plastic zip-lock bags at depth and brought to the surface, then placed in seawater filled coolers and transported back to the laboratory (<3 h). Clean seaweeds, free of substantial epiphyte growth or other macroscopic material, were frozen at −20°C. Samples of the green alga *H. tuna* were flash-frozen in liquid nitrogen to prevent degradation of the diterpenoid compounds [42]. All frozen algal samples were transported on ice to the Smithsonian Marine Station in Fort Pierce, FL for chemical extraction and analysis. These macroalgae were chosen based on their abundance on Caribbean reefs and their previously identified chemical activity against coral reef-associated bacterial cultures [40].

**Table 1:** Natural Concentrations of algal extract (mg)/algal wet wt.(g).

Florida 2009	Location (Year) Algae Collected	Concentration (mg/g)*	Experiment Deployed	Samples Retrieved
*H. tuna* (organic)	Florida Keys (2008)	12.02	5/23/09	5/26/09
*L. variegata* (organic)	St. Thomas (2008)	16.51	5/23/09	5/26/09
*L. variegata* (aqueous)	St. Thomas (2008)	29.9	5/23/09	5/26/09
**Belize 2009**				
*Dictyota* sp. (organic)	Belize (2008)	7.53	8/7/09	8/10/09
*H. tuna* (organic)	Belize (2006)	24.4	8/7/09	8/10/09
*L. variegata* (organic)	St. Thomas (2008)	16.51	8/14/09	8/17/09
*L. variegata* (aqueous)	St. Thomas (2008)	29.9	8/14/09	8/17/09

* Concentration at which extracts were incorporated into treatment gels.

Extraction of compounds from macroalgal thalli was conducted at the Smithsonian Marine Station in Fort Pierce, FL. Frozen bulk samples of macroalgae were first wet-weighed and then lyophilized until dry. Lyophilized samples were weighed and exhaustively extracted with a 1∶1 ethyl acetate:methanol (organic or non-polar extract) solvent solution over 3 consecutive 24-h periods, followed by three 24-h extractions in 1∶1 ethanol:deionized water (aqueous or polar extract). Extracts were filtered and the solvents removed via rotary evaporation at 35°C followed by solvent removal in a Thermo-Savant speed-vac concentrator. Dry extracts were stored frozen (−20°C) and transported on ice to field laboratories for extract experiments. We tested extracts at natural concentrations based on algal wet weight (g) (Table 1), similar to methods used by [43].

### Algal Extract Assay

Assay techniques were adapted from a previous study that examined the effect of sponge extracts on coral photosymbionts [44]. Experiments were conducted on both *Montastraea faveolata* and *Porites astreoides* corals at 10–15 m depth on Wonderland Reef (N 24′ 33.623 W 81′ 30.082) adjacent to Summerland Key, FL (May 2009) and on South Water Cay Reef (N 16′ 49.168 W 88′ 04.677) in Belize (August 2009). Extracts were dissolved in 5-ml of 95% ethanol and incorporated into polysaccharide gels (3 g of Phytagel +180 ml of DI water to make 10 plates) at natural concentrations and poured into 5-cm plastic petri dishes leaving the gel surface 2–3 mm below the top of the petri plate. The outside of each dish was covered with duct-tape to maintain consistent light levels when applied to the coral surface. Holes were drilled into opposite sides of the petri dish and a zip-tie threaded through, to which a bungee cord was attached.

**Figure 1:**
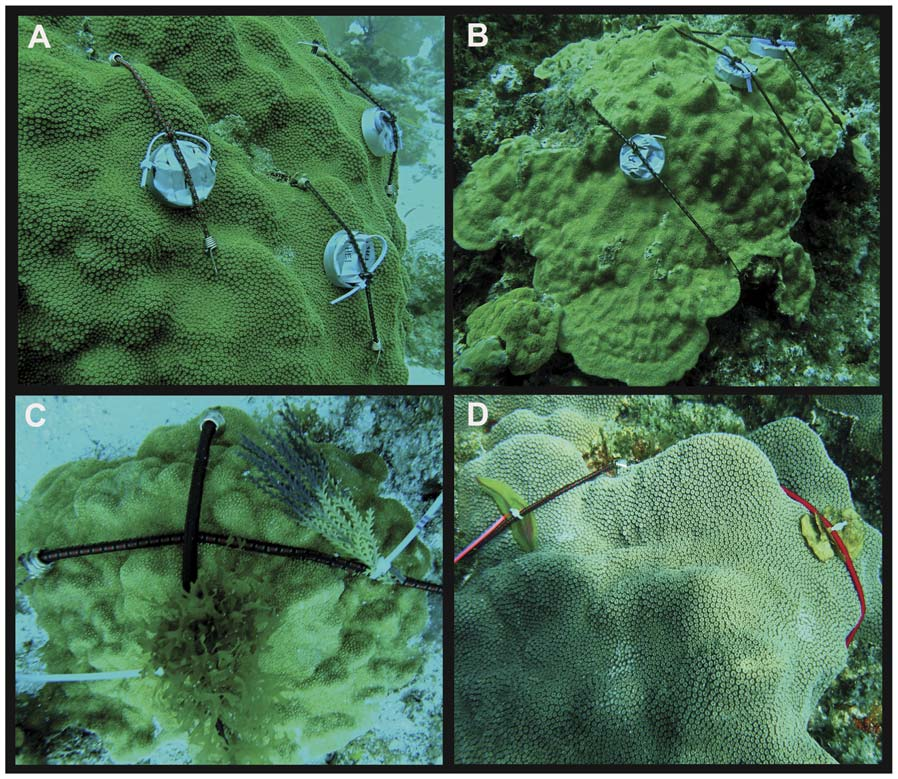
Photos of extract and algal application. Photos illustrate three experimental plates (shade control, solvent control, and macroalgal extract) on: A. *M. faveolata* and B. *P. astreoides* corals. Experimental application of C. *Dictyota* sp. foliose brown macroalgae and algal mimic to a *P. astreoides* coral colony in Belize (2009), and D. *Lobophora variegata* decumbant brown macroalgae and algal mimic to a *M. faveolata* coral colony in Belize (2009).

Immediately after the gels had solidified (<1 hr), SCUBA divers attached each petri plate such that the gel surface faced the coral colony. The gel remained ∼2 mm above the colony surface to prevent smothering of coral tissues and to allow coral tentacles to contact the gel when extended. Petri plates were fixed in place by stretching the attached bungee cord around coral colonies and tapping each bungee cord hook into the coral skeleton or hooking around the colony edge (Fig. 1A & 1B; all corals recovered in <1 wk from bungee cord attachment). For each experimental colony (n = 5), three petri plates were attached: 1) shading/plate control with no gel, 2) solvent (EtOH) control with gel but no extract, and 3) treatment gel containing macroalgal extract. Immediately prior to petri-plate attachment 5-ml plastic tip syringes were used to collect triplicate samples from the coral surface mucus layer (SML) (5×5 cm area) to assess the initial microbial assemblage. Each syringe was capped pre- and post-sample collection to prevent additional seawater contamination, and sterile nitrile gloves were worn to reduce human bacterial contamination. For each extract five healthy coral colonies of each coral species served as the replicates in Florida and Belize.

Deployed petri plates were monitored daily when possible to ensure they were undisturbed and remained in place. After ∼72 hrs of exposure, bacterial samples were collected, as described above, from areas of the coral SML not exposed to petri-plates (n = 3 per coral). A 5-ml microbial sample was also collected from the SML directly under each petri-plate treatment (Fig. 1). After samples were brought to the surface, syringes were placed in seawater-filled coolers and transported back to the laboratory (<3 hrs), where they were immediately processed for transport and subsequent culture-independent analyses. Syringes were placed tip down in test-tube racks for ∼15 minutes to allow the mucus to settle to the bottom, then 2 ml of concentrated mucus was transferred to cryovials and centrifuged at 10,000×*g* for 10 min. The seawater supernatant was poured off and the remaining mucus pellet frozen at −20°C. Microbial samples were transported back to Auburn University on ice and thawed at 4°C prior to DNA extraction using the MOBIO Ultraclean® Microbial DNA Isolation kit (Carlsbad, CA), according to the manufacturer’s instructions, with an additional (10 min) heating step to 64°C to increase DNA yield. Extracted DNA was stored at −80°C until PCR amplification.

### Cellular Stress Enzyme Assays

After syringe samples were taken for bacterial analysis, a small (1 cm^2^) sample of coral tissue was chiseled off of each colony from under each petri-plate treatment, and from a location on the colony that was not exposed to treatments. Tissue samples were immediately returned to the field laboratory, flash frozen in liquid N_2_, transferred to the University of North Florida on dry ice and stored at −80°C. Tissue samples were only taken in Belize during August 2009, and not in Florida.

Samples were thawed to room temperature and extracted in 2.5 mL of buffer (50 mM potassium phosphate buffer (pH 7.0) containing 10% (w/v) polyvinylpolypyrrolidone (PVP)-40, 0.25% Triton X-100, and 1% (v/v) plant cell protease inhibitor cocktail [Sigma–Aldrich, St. Louis, MO, USA]. Samples were homogenized with a Fast Prep 24 bead homogenizer (MP Biomedicals, Irvine, CA, USA) and centrifuged at 16,000×g for 10 min. The resulting supernatants were normalized for protein content using a Bicinchoninic acid (BCA) Protein Assay Kit (Thermo Scientific, Pittsburgh, PA, USA).

To monitor levels of induced oxidative stress, superoxide dismutase (SOD) and catalase (CAT) activities were used as cellular proxies. Up-regulation of SOD and CAT activities reflects an organism’s response to counteract the presence of damaging reactive oxygen species. Superoxide dismutases are considered ubiquitous metalloenzymes that catalyze the dismutation of the reactive superoxide anion (O_2_^−^) into H_2_O_2_ [45]. SOD activity was measured using a Superoxide Dismutase Assay Kit (Cayman Chemical). Catalase, another widely distributed enzyme that destroys H_2_O_2_, by dismutation to O_2_ and H_2_O [46], was assayed using the Amplex Red kit (Invitrogen, Eugene, OR, USA) according to the manufacturer’s instructions.

To further assess the response of corals to algal secondary metabolites, the detoxification enzyme glutathione-S-transferase (GST) was measured. GSTs play a key role in the cellular detoxification of xenobiotics [47] and damaging lipid hydroperoxides [48]. A GST activity kit (Cayman Chemical, Anne Arbor, MI, USA) was used according to the manufacturer’s instructions. Differences in enzyme concentrations for each coral species were analyzed by one-way ANOVA as a function of treatment followed by Tukey post-hoc tests run in the R statistical package [49].

### Live Algal Application

Live samples of the brown macroalgae *L. variegata* and *Dictyota* sp. were collected at South Water Cay reef in Belize and kept in running seawater tables overnight. Thalli of approximately the same weight (see results for mean weights) were gathered and attached to bungee cords with small zip-ties. Algal mimics (i.e. plastic aquarium plants) of similar size and shape to live macroalgae were also attached to bungee cords with zip-ties and left in running seawater tables overnight. Using methods similar to those outlined above, live macroalgae and algal mimics were attached to both *M. faveolata* and *P. astreoides* corals at ∼8–15 m depth on South Water Cay Reef, Belize (Fig. 1C & 1D). As described above, triplicate samples were taken from the coral SML prior to algal application. After 72 hrs. of exposure, another set of triplicate SML samples were taken in addition to a microbial sample from under the live macroalgae and algal mimics. Samples were transported and processed as outlined above. All macroalgae and algal mimics were weighed after the experiment was completed. Unfortunately, the majority of samples collected from *P. astreoides* corals were lost, thus we only report results from the *M. faveolata*-algal interaction experiment.

### PCR Amplification and DGGE Analysis

Universal bacterial primers 27F-GC (5′ -CGC CCG CCG CGC GCG GCG GGC GGG GCG GGG GCA CGG GGG CAG AGT TTG ATC MTG GCT CAG-3′) and 518R (5′ -ATT ACC GCG GCT GCT GG-3′), were used to amplify the 16S rRNA gene from bacterial isolated genomic DNA from the coral SML. The forward primer was modified to incorporate a 40-bp GC *clamp* for resolution on a denaturing gradient gel electrophoresis (DGGE) system [50], [51]. These primers amplify a 491-bp section of the 16S rRNA gene of members of the domain *Bacteria*, including the highly variable V1–V3 regions [52], [53]. All PCR was performed on a thermal cycler (model: Master cycler epgradient, Eppendorf, Hauppauge, NY) as follows: 12.5 µl EconoTaq PLUS GREEN 2X Master Mix (Lucigen), 0.5 µl of each 20 µM primer, and adjusted to a final volume of 25 µl with nuclease-free water. Strip tubes, master mix and nuclease free water were UV-irradiated for 20 minutes prior to the addition of primers under sterile conditions in a laminar flow hood to reduce contamination [54]. DNA template was added during an initial ‘hotstart’ of 3 min at 94°C, followed by a ‘touchdown’ PCR protocol, in which the annealing temperature was decreased from 65°C by 1°C every cycle until reaching a touchdown temperature of 54°C, at which temperature 35 additional cycles were performed as follows; 94°C for 45 sec, 54°C for 45 sec, and 72°C for 1.5 min; followed by a final extension at 72°C for 7 min. PCR products were analysed by agarose gel electrophoresis (1% w/v agarose) stained with ethidium bromide and visualized using a UV transilluminator.

Samples were separated using a Hoefer SG50 (Hoefer Inc.) DGGE system. PCR products were loaded onto an 8% acrylamide gel and run with 0.5 X TAE buffer (Tris base, acetic acid, EDTA) and a 35–60% linear denaturing gradient of formamide and urea. Gels were electrophoresed at 60°C for 15 min at 50 V, and subsequently for 10 hrs at 100 V (1000 V·hrs) on the DGGE system. After electrophoresis the gels were stained for 30 min with 1X SYBR-Gold nucleic acid stain (Invitrogen) in 0.5 X TAE buffer, rinsed, and photographed using a UV transilluminator (AlphaImager, Cell Biosciences). Images were saved as 8-bit TIFF files followed by alignment, normalization, band class identification, and statistical analyses using Bionumerics V. 5.0 (Applied Maths).

### Band Excision and Sequencing

Uniquely dominant and distinct bands were dabbed with a sterile pipette tip and placed directly into PCR strip tubes containing UV-sterilized nuclease free water. Bands were re-amplified with the previously described touchdown protocol using the 27F/518R primer set without the -GC clamp. PCR products were analyzed with agarose gel electrophoresis (1% w/v agarose) stained with ethidium bromide and visualized using a UV transilluminator. An ammonium acetate-ethanol precipitation was performed and the resulting product amplified using the BigDye® sequencing reaction; 1.0 µl of BigDye®, 1.5 µl of 5x Buffer, 0.5 µl of 10 µM 27F, 4 µl of nuclease free water, 3 µl of Template. The following thermal cycler conditions were used; 95°C for 30 s, 50°C for 30 s, and 60°C for 4 min, at which temperature 30 additional cycles were performed. PCR products were purified using the BigDye® XTerminator Purification Kit (Applied Biosciences) and shipped to the Smithsonian Institution Laboratories of Analytical Biology in Suitland, MD for sequencing. The Smithsonian Institution performed high-throughput (96-well) sequencing on an ABI sequencer. Sequences were trimmed using CLC Genomics Workbench (CLC Bio) and compared to the NCBI nr/nt GenBank database by BLASTn algorithm and those sequences with >96% identity and E-values <1×10^−20^ were accepted for downstream analysis.

### DGGE Profile Analysis

DGGE images were imported into Bionumerics V 5.0 (Applied Maths) and processed to allow multiple gel images to be reliably compared, including: identify sample lanes, apply a background subtraction, normalize to the reference standards run on each gel, and identify and quantify bands. Sample comparison and band matching was initially conducted in Bionumerics, in which band classes were constructed based on optimal position tolerance and optimization settings. A bifurcating hierarchical dendrogram and similarity matrix representing sample clusters was constructed for each coral-extract interaction at each site using the WARD algorithm and DICE coefficients derived from the band alignment. A binary matrix based on band presence/absence was exported from Bionumerics, imported into the R statistical package and converted to a distance matrix for statistical analysis [49].

To characterize any changes in the bacterial community Kruskal’s nonmetric multidimensional scaling (nMDS) analysis and permutational multivariate analysis of variance (PERMANOVA) were used to assess the multivariate relationships among and between binary DGGE profiles for each coral-extract species pair at each site (Florida & Belize). A significant PERMANOVA result (P<0.05) suggests that initial coral microbial assemblages are significantly different from extract- and/or control-associated microbial samples. The Euclidean distances (we used Jaccard) between points in an nMDS plot are inversely proportional to the similarity of the samples. The number of dimensions (*k*) was determined by first running a scree plot to determine stress (i.e. an inverse measure of fit to the data) as a function of dimensionality. Kruskal’s stress formula was used as an informal method of determining the appropriate number of dimensions [55]. These data were analyzed using the metaMDS and Adonis utilities within the Vegan package in R. metaMDS is unique in that it calls on isoMDS to perform nMDS, but also searches for the most stable solution by performing several random starts (we used 20; [49]). The relationship among samples is represented in a plot of the first two nMDS dimensions.

A final analysis of the data was completed using Multi-response Permutation Procedures (MRPP), within the Vegan package in R. We combined the three post-treatment controls (shading, solvent, and post SML) into a ‘treatment controls’ group. Thus, MRPP determined whether the 3 sampling treatments (initial SML, treatment controls, and extract treatment) formed distinct groupings based on their DGGE profiles. In an MRPP analysis samples are *a priori* assigned to 2 or more groups (in our case 3 groups) and used to calculate a distribution of the average intra-group distance by randomly permutating the data *n* times (in our case *n = *5000), which determines a *p*-value for the observed intra-group distance in the data. Additionally, an *A* parameter is calculated that relates the observed intra-group average distance to the mean of the calculated distribution. When the value of *A* is close to 1 it is indicative of very tight groupings. We performed MRPP separately on each of 14 coral-extract pairs, *M. faveolata* and *P. astreoides* corals paired with *H. tuna* (organic) and *L. variegata* (organic and aqueous) at both sites and with *Dictyota* sp. (organic) in Belize.

## Results

### Coral Bacterial Assemblages

**Figure 2:**
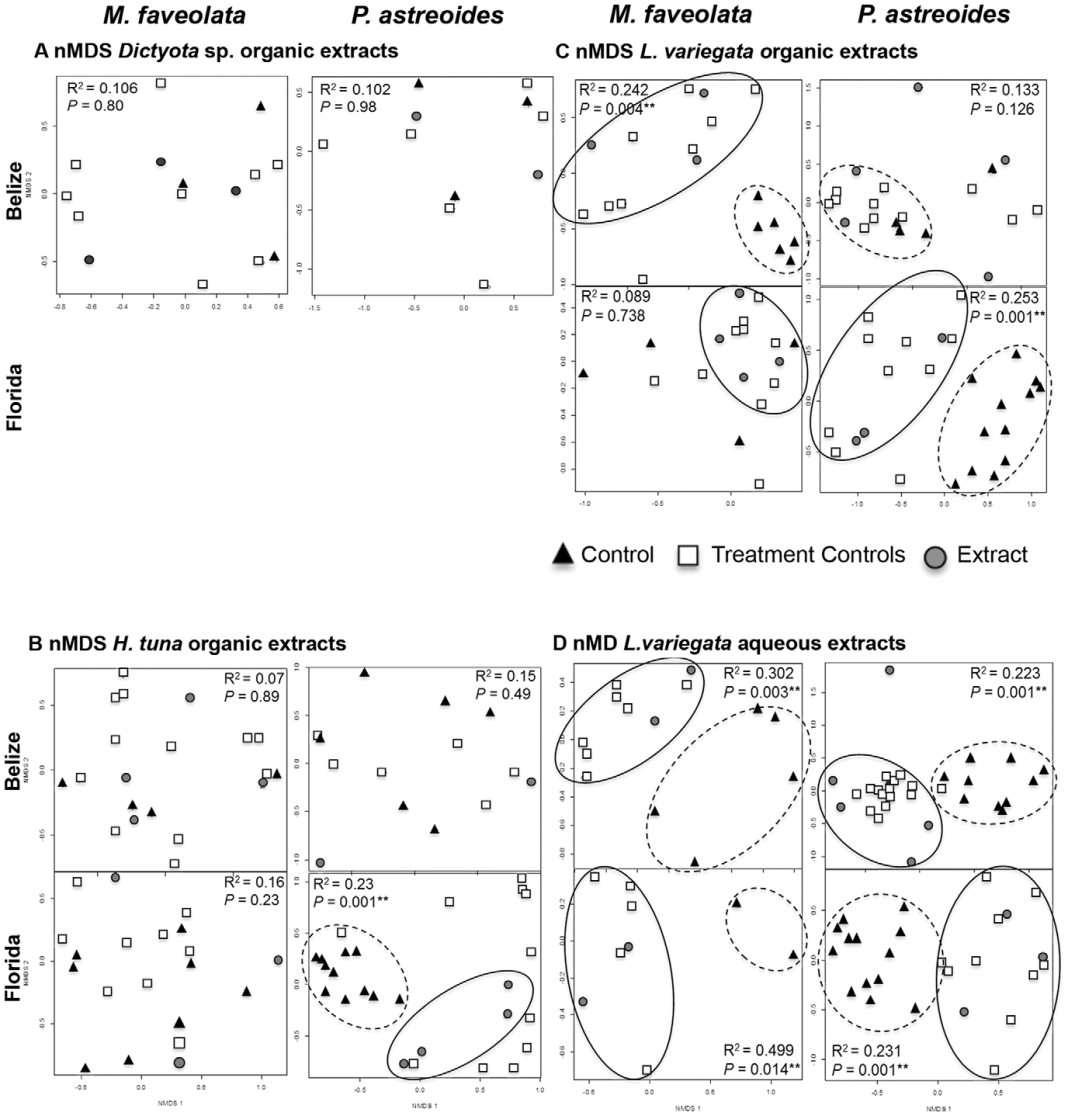
nMDS plots and PERMANOVA results. nMDS plots based on binary DGGE profiles for each coral-extract interaction pair at each site (Belize & Florida) between *M. faveolata* or *P.astreoides* corals and extracts of: A. *Dictyota* sp. (organic), B. *H. tuna* (organic), C. *L. variegata* (organic), and D. *L. variegata* (aqueous). Solid circles enclose clusters related to extract-associated microbial samples and dashed circles enclose clusters related to initial coral control-associated microbial samples. PERMANOVA results are reported in the corner of each interaction pair, ** indicates a significance of P<0.05.

We examined the effect of four macroalgal extracts on the bacterial assemblages associated with the SML of *M. faveolata* and *P. astreoides* corals in Belize and Florida using PCR-DGGE. DGGE profiles based on 16S rRNA universal bacterial primers (27F/518R) revealed diverse bacterial assemblages for all samples with intraspecific similarity among initial controls. Extracts caused a range of effects on coral-associated bacteria, but generally increased in activity: *Dictyota* sp. < *H. tuna* < *L. variegata* (organic) < *L. variegata* (aqueous). Analysis of DGGE profiles using nMDS and PERMANOVA suggest that neither *Dictyota* sp. (organic) extracts nor treatment controls had a significant effect on bacterial assemblages within the SML of either coral species (Fig. 2A; Table 2A and B). These results illustrate that the physical manipulation of the experiment, including the controls for shading and solvent, did not significantly alter coral bacterial assemblages and that not all extracts, which are rich in both primary and secondary metabolites, had an effect.

**Table 2:** PERMANOVA and MRPP results for algal extract application and live algal thalli application.

a. PERMANOVA					
Belize		*Dictyota* sp. (organic)	*H. tuna* (organic)	*L. variegata* (organic)	*L. variegata* (aqueous)
*M. faveolata*	R^2^	0.106	0.07	**0.242**	**0.302**
	P	0.804	0.887	**0.004** **	**0.003** **
*P. astreoides*	R^2^	0.102	0.151	0.133	**0.223**
	P	0.98	0.492	0.126	**<0.001** **
**Florida**					
*M. faveolata*	R^2^	–	0.158	0.089	**0.499**
	P	–	0.233	0.738	**0.014** *
*P. astreoides*	R^2^	–	**0.229**	**0.253**	**0.231**
	P	–	**<0.001** **	**<0.001** **	**<0.001** **
**b. MRPP** a					
**Belize**		***Dictyota*** ** sp. (organic)**	***H. tuna*** ** (organic)**	***L. variegata*** ** (organic)**	***L. variegata*** ** (aqueous)**
*M. faveolata*	A	−0.033	−0.064	**0.076**	**0.093**
	Δ	0.863	0.996	**0.006** **	**0.002** **
*P. astreoides*	A	−0.073	−0.011	0.022	**0.099**
	Δ	0.967	0.669	0.114	**<0.001** **
**Florida**					
*M. faveolata*	A	–	0.004	−0.013	**0.167**
	Δ	–	0.35	0.709	**0.024** *
*P. astreoides*	A	–	**0.092**	**0.098**	**0.087**
	Δ	–	**<0.001** **	**<0.001** **	**<0.001** **
**c. Live Algae**					
***M. faveolata***		***Dictyota*** ** sp. (live algae)**			***L. variegata*** ** (live algae)**
**PERMANOVA** b	R^2^	0.135	–	–	**0.236**
	P	0.896	–	–	**0.006** **
**MRPP** b	A	0.039	–	–	**0.048**
	Δ	0.926	–	–	**0.005** **

a Function of initial controls, experimental controls (solvent + shade + post controls combined), and treatment extracts,

b Function of initial controls, post controls, algal mimic, live algae,

* P<0.05,

** P<0.001.

The organic extracts from *Halimeda tuna* did induce a shift in the bacteria associated with *P. astreoides* corals (P<0.001) in Florida, based on PERMANOVA/MRPP results (Table 2) and nMDS plots (Fig. 2B). However, the same extract did not change the bacterial assemblages associated with either coral in Belize (Fig. 2B). The effect may not have been detected in Belize due to low replication, since only two of the five sample sets were complete after collection and extraction.

Bacterial samples from *M. faveolata* corals exposed to organic extracts from the brown alga *L. variegata* were significantly altered in Belize (PERMANOVA; P = 0.004), but not in Florida (P = 0.738; Table 2), when compared to initial controls. The opposite was true for *P. astreoides* corals; a significant change was detected on Florida corals (PERMANOVA; P<0.001), but not in Belize (P = 0.126; Table 2). Based on nMDS plots (Fig. 2C) and MRPP results (Table 2B), the entire colony bacterial assemblages were shifted by *L. variegata* (organic) extracts on *M. faveolata* corals in Belize and on *P. astreoides* corals in Florida. The extract primarily had a point-source effect in Florida *M. faveolata-L. variegata* (organic) interactions, directly under the applied extract, and not on the surrounding colony bacteria (P = 0.738; Table 2). There was no effect of *L. variegata* (organic) extracts on *P. astreoides* in Belize, most samples clustered together in the nMDS analysis (Figure 2C).

*L. variegata* (aqueous) extracts had extensive effects on all coral-bacterial assemblages at both sites. Results show that *L. variegata* (aqueous) extracts altered the entire coral colony microbiota, including all treatment controls (shade & solvent) and post-SML samples, to a bacterial assemblage that was significantly different in structure from initial SML bacteria (PERMANOVA; P<0.01; Table 2; Fig. 2D). Similarity results based on hierarchical cluster analyses showed that *P. astreoides* post treatment samples were ∼36% similar to initial samples from the same colonies, and *M. faveolata* post treatment samples were ∼7% similar to initial samples from the same colonies.

**Figure 3:**
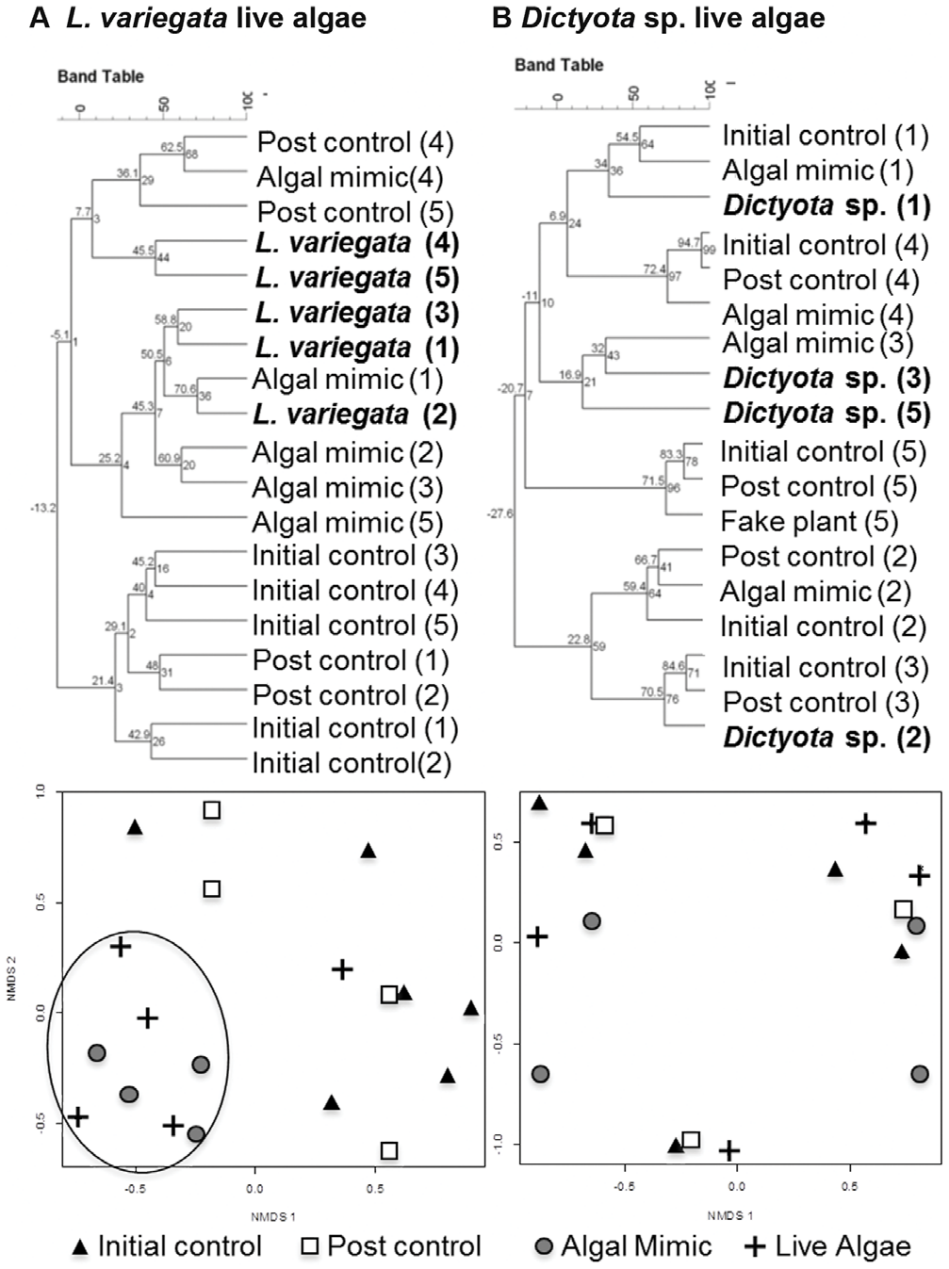
Results for live algae application. Hierarchical cluster analyses showing similarity among microbial samples (%; inner tree) with bootstrap support (1000 iterations, outer tree) to examine the effect of live macroalgae in the genera: A. *L. variegata* and B. *Dictyota* sp. applied to *M. faveolata* coral colonies in Belize 2009. Numbers in parentheses correlate to the experimental colony (#1–5). Results are further illustrated by nMDS plots of each coral-algal pair.

In Belize whole algal thalli of *L. variegata* and *Dictyota* sp. were applied to *M. faveolata* colonies, which had similar results to the extract application experiment. Thalli of *L. variegata* macroalgae that weighed 1.5 g ±0.3 SE and algal mimic controls, which weighed 1.4 g ±0.2 SE, were applied to coral colonies (n = 5). Hierarchical cluster analyses and nMDS plots (Fig. 3A), in addition to PERMANOVA results (P = 0.006; Table 2C) illustrate that samples taken from under both live *L. variegata* and algal mimics cluster together and are significantly different from initial and post controls. Thalli of *Dictyota* sp. that weighed 0.5 g ±0.1 SE and algal mimics that weighed 1.2 g ±0.2 SE were also applied to *M. faveolata* colonies in Belize. Hierarchical cluster analyses showed no effect of live *Dictyota* sp. to coral bacterial assemblages (Fig. 3B). Most bacterial samples (initial control, post control, macroalgae, and algal mimic) clustered by coral colony (Fig. 3B). These data were analogous to the reported extract results and supported by nMDS plots (P = 0.896; Fig. 2A) and PERMANOVA results (Table 2), which demonstrated little effect of *Dictyota* sp. extract on *M. faveolata* coral-bacterial assemblages.

### Bacterial Identity

Of the 150 bands excised and sequenced from DGGE gels only 19 sequences were of sufficient quality to be identified (>96% identity) from SML samples of *M. faveolata* and *P. astreoides* coral colonies (Table 3). Sequences from initial SML samples closely matched the common bacteria found in a previous 454-pyrosequencing study (see [7]) that examined bacterial assemblages associated with natural coral-algal interactions.

**Table 3:** 16S rRNA sequence identification (>96% identity ) of excised DGGE bands.

Coral Species	Initial Control
***M. faveolata***	*α-proteo; Rhodobacterales; Rhodobacter* (n = 1)
	*γ-Proteo; Enterobacterales*; *Edwarsiella* (n = 2)
	*γ-Proteo; Oceanospirillales; Halomonas* (n = 1)
***P. astreoides***	*γ-Proteo; Enterobacterales*; *Edwarsiella* (n = 2)
	*γ-Proteo; Oceanospirillales; Halomonas* (n = 1)
	*Firmicutes; Bacillales; Bacillus* (n = 1)
	**Extract Treatment**
***M. faveolata***	*Cyanobacteria (uncultured)* (n = 1)
	*Cyanobacteria; Synechococcus* (n = 2)
	*Firmicutes; Bacillales; Bacillus* (n = 1)
	*Actinobacteria* (n = 1)
***P. astreoides***	*γ-Proteo; Enterobacterales; Edwarsiella* (n = 2)
	*γ-Proteo; Oceanospirillales* (n = 1)
	**Experimental controls (solvent, shade, post)**
***M. faveolata***	*γ-Proteo; Oceanospirillales* (n = 1)
***P. astreoides***	*γ-Proteo; Oceanospirillales* (n = 1)
	*γ-Proteo; Oceanospirillales; Halomonas* (n = 1)
	*γ-Proteo; Methylococcales; Methylomonas* (n = 1)

### Cellular Stress Enzyme Assays

**Figure 4:**
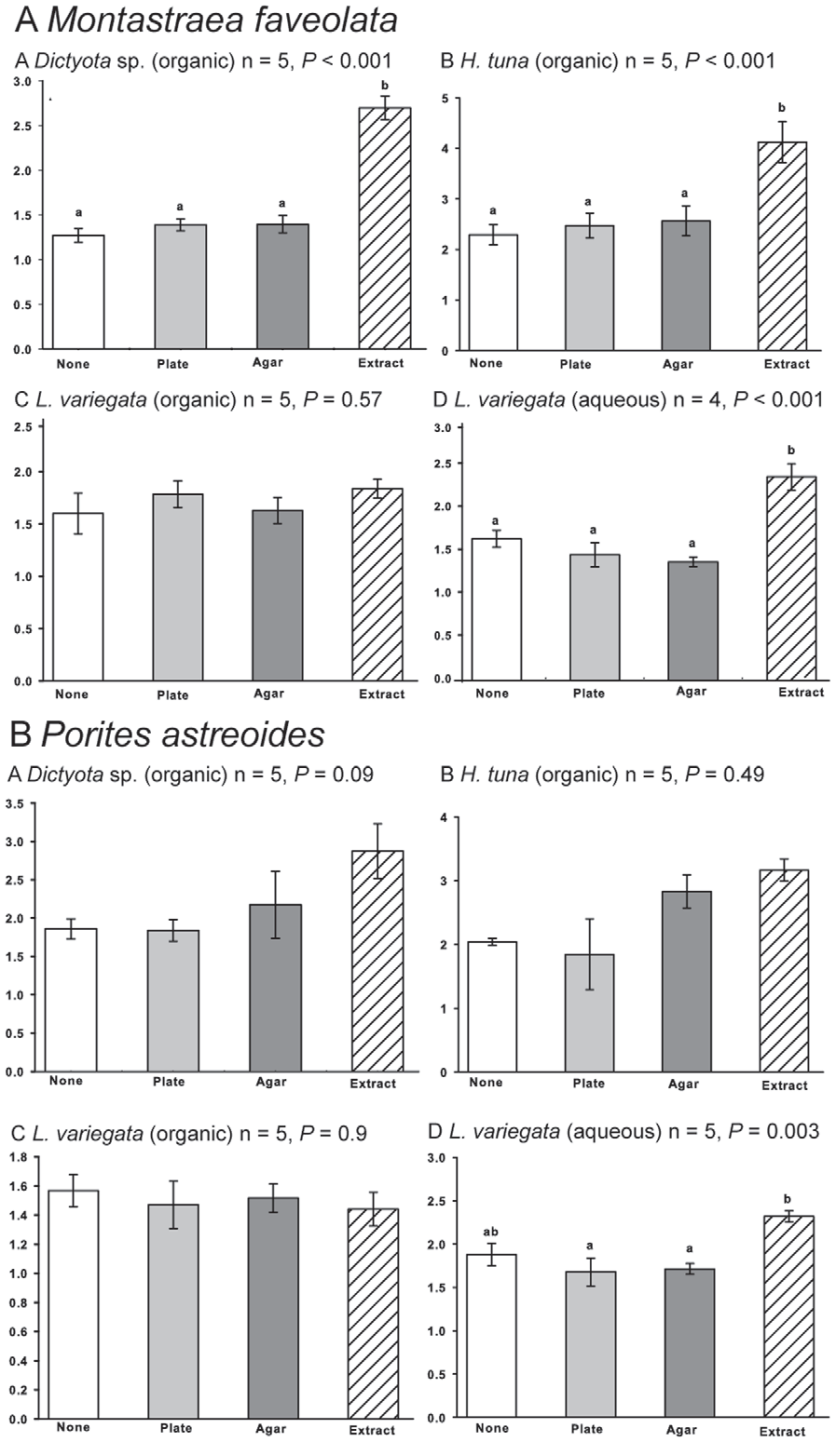
GST activity for corals exposed to extracts. Glutathione S-transferase (GST) activity of A. *M. faveolata* and B. *P. astreoides* corals exposed to macroalgal extracts following a 72 h exposure period: Application of A. *Dictyota* sp. organic extract, B. *Halimeda tuna* organic extract, C. *Lobophora variegata* organic extract, D. *Lobophora variegata* aqueous extract for each coral species. GST activity is expressed in nmol min^−1^ mg protein ^−1^.

Following the 72-h exposure period to algal extracts, SOD and CAT activities were not observed to change regardless of treatment (Table 4). However, when *P. astreoides* samples were treated with live *Lobophora variegata* a significant decrease in SOD (p = 0.005) and CAT (p = 0.043) activity was detected when compared to controls. Significant changes in the activity of GST in *M. faveolata* were detected when samples were exposed to *Dictyota* sp. (organic) extract (p<0.001); *H. tuna* (organic) extract (p<0.001); and *L. variegata* (aqueous) extract (p<0.001) (Fig. 4A). Only *L. variegata* (aqueous) extracts caused a significant change in *P. astreoides* GST activity (Fig. 4B). This change was detected in both the tissues beneath the extract treatments and in adjacent control tissues. The organic extract of *L. variegata* had no effect on CAT, SOD or GST activities in any of the corals tested.

**Table 4:** SOD and CAT concentration results for experiments conducted in Belize (2009).

Coral Species	Algal Treatment^a^	SOD activity (U/mg protein)^b^			CAT activity (U/mg protein)^b^		
		Control^c^	Treatment	*P* value	Control	Treatment	*P* value
***M. faveolata***	*Dictyota* sp. (org.)	0.42±0.0	0.49±0.1	0.938	0.21±0.0	0.19±0.1	0.719
***''***	*Dictyota* sp. (live)	0.54±0.1	0.70±0.1	0.387	0.20±0.1	0.30±0.1	0.453
***''***	*L. variegata* (aqua)	1.57±0.5	1.42±0.2	0.415	0.24±0.1	0.44±0.2	0.588
***''***	*L. variegata* (org.)	1.61±0.2	1.50±0.3	0.968	0.32±0.1	0.37±0.1	0.979
***''***	*L. variegata* (live)	1.19±0.3	0.82±0.1	0.114	0.69±0.2	0.42±0.1	0.4
***''***	*H. tuna* (org.)	0.68±0.1	0.74±0.1	0.433	0.28±0.8	0.36±0.1	0.323
***P. astreoides***	*Dictyota* sp. (org.)	0.59±0.1	0.61±0.1	0.924	0.41±0.1	0.34±0.1	0.776
***''***	*Dictyota* sp. (live)	1.14±0.0	0.89±0.2	0.72	0.61±0.1	0.50±0.1	0.799
***''***	*L. variegata* (aqua)	1.74±0.2	1.13±0.2	0.215	0.32±0.1	0.54±0.2	0.582
***''***	*L. variegata* (org.)	1.36±0.4	1.71±0.3	0.594	0.48±0.2	0.29±0.1	0.777
***''***	*L. variegata* (live)	0.95±0.1	0.49±0.1	0.005	0.67±0.1	0.36±0.0	0.043
***''***	*H. tuna* (org.)	0.64±0.0	0.93±0.2	0.386	NA	NA	NA

a Algal treatments are organic (org.), aqueous (aqua), or live algal thalli (live) applications.

b Mean concentration ± SE in coral tissue after 3 day exposure to treatments (n = 5). *P*-values generated with one-way ANOVA and Dunnett post-hoc tests.

c Control values are collected from coral samples under the solvent plate control.

## Discussion

Benthic macroalgae can compete with corals using multiple mechanisms, and the data presented here demonstrate that allelopathy can cause sublethal stress and distinct shifts in the bacterial communities associated with corals. Macroalgal allelochemicals have been shown to inhibit microbes and control pathogens or epiphytes [35], [40], [43]. These macroalgal allelochemicals include fatty acids, phenolics, acetylenes, terpenes, coumarins, and polysaccharides (reviewed in [41]). Until recently, only a few studies had examined the role of these compounds in natural systems [35], [43], but it is believed that metabolites derived from macroalgae selectively target marine microorganisms [35], and that their concentrations can increase during microbial attack [56]. This is the first *in situ* study to show the dynamic effect of macroalgal compounds on coral- associated bacteria.

Bacterial communities within the surface mucus of *M. faveolata* and *P. astreoides* colonies were significantly altered from initial communities by some macroalgal extracts on both Florida and Belize reefs. The extent to which extracts shifted the bacterial assemblages depended on both the coral host and type of macroalgae tested. We also demonstrate that there was a significant increase in glutathione S-transferase activity (GST), an enzyme critical for the anti-oxidant response and detoxification of potentially harmful compounds, within coral tissues exposed to three of the four macroalgal extracts. Large shifts in the bacterial assemblages along with heightened coral stress responses suggests that *M. faveolata* is more susceptible to macroalgal allelopathy than *P. astreoides*. This may provide further indication that *P. astreoides* corals are more robust than *M. faveolata,* a genera that is also susceptible to a larger number of diseases than *P. astreoides* [57], [58]. Visual signs of degradation or bleaching were never observed, suggesting that the experimental treatments were not smothering the coral nor were the extracts visibly affecting *Symbiodinium* sp. in the coral tissues.

Macroalgal extracts and live algal thalli from *Dictyota* sp. had the least effect on coral bacterial assemblages. Negative results with these extracts demonstrate that the treatment itself (e.g. shading, exposure to solvent and algal terpenes, manipulation), did not cause detectable changes to the coral-associated bacteria. Thus, the changes identified in the bacterial assemblages during concurrent and subsequent experiments were most likely a result of the composition of the applied extracts. From an organismal perspective, *Dictyota* sp. extracts did cause a significant increase in GST levels in *M. faveolata* corals, but not *P. astreoides.* Therefore, extracts from *Dictyota* sp. can cause sublethal stress to *M. faveolata* tissues that are in direct contact, regardless of changes to the bacterial community. We chose *Dictyota* sp. brown macroalgae because members of this genus are dominant members of the benthos throughout the Caribbean [59]. They also produce terpenes active against herbivory and biofouling [60], [61], organic metabolites that damage corals by causing bleaching and decreased photosynthetic efficiency when in direct contact [15], [17]. Finally, two species of *Dictyota* algae and one crude extract reduced the survival and settlement of *P. astreoides* larvae [14].

Green calcareous macroalgae in the genus *Halimeda* are also commonly found throughout the Caribbean, Tropical-Pacific, and Indian Ocean [59], [62]. Most species produce activated defenses in the form of diterpenoid compounds [42], quickly converting halimedatetraacetate to the more potent defensive compound halimedatrial upon damage. We tested all *Halimeda* extracts used in this study and found that they only contained halimedatetraacetate, not halimedatrial, as expected since the alga had been frozen in liquid nitrogen before extraction to prevent activation and degradation of compounds. Thus, our results are conservative compared to the possible effects of live macroalgae in the field. The only significant bacterial shift due to *H. tuna* extract exposure was associated with *P. astreoides* corals in Florida. However, sublethal stress was detected in *M. faveolata* coral tissues and not *P. astreoides* exposed to *H. tuna* extracts. Thus, extracts can cause tissue stress without altering bacterial assemblages in the surface mucus layer and vice versa. We previously demonstrated that cultured coral reef bacteria varied widely in their response to *H. tuna* extracts, which caused both bacterial growth and inhibition [40]. It is possible that the bacteria associated with *M. faveolata* are less affected by *H. tuna* extracts than those associated with *P. astreoides,* but further research is needed to confirm these differences. Examining the bacterial response within stressed coral tissues would be a logical next step.

The most distinct changes to coral bacterial assemblages were in response to polar compounds and live algal thalli from the brown macroalga *L. variegata.* Bacterial samples from both *M. faveolata* and *P. astreoides* coral colonies were dramatically shifted as a result of *L. variegata* (aqueous) extract exposure. All surface mucus samples, including shading and solvent controls, were shifted to a completely different and distinct assemblage of bacteria in comparison to initial controls. This unprecedented result suggests that a relatively small area of exposure (5-cm diam. gel) to extracts over a short period of time (3 days) may have colony-wide effects on coral-associated bacteria. Furthermore, GST levels were significantly higher in both *M. faveolata* and *P. astreoides* corals exposed to *L. variegata* (aqueous) extracts. This was the only extract to cause sublethal tissue stress to *P. astreoides* colonies, not only under the extract but in the adjacent tissues as well. Corals exposed to live *L. variegata* thalli also experienced a change in the bacterial community; however the shift was not colony wide and also occurred under the algal mimics and post controls in some cases. Similar results between the live algal thalli, algal mimics, and post controls suggest that *L. variegata* thalli can cause colony-wide changes in bacterial assemblages. However, shifts under the algal mimic suggest that shading and abrasion may also play a role in the effect that *L. variegata* has on coral-associated bacteria, but additional replication is needed to test this hypothesis.

Organic extracts from *L. variegata* did not cause as profound a shift in bacterial assemblages as the aqueous extracts, nor were the results consistent across sites. It appears that *L. variegata* (organic) extracts caused a significant bacterial shift within the SML of *P. astreoides* corals only in Florida, and within the SML of *M. faveolata* corals only in Belize. The non-significant results for *M. faveolata-L.variegata* (organic) interactions in Florida in comparison to Belize is likely due to more variable bacterial assemblages within the initial control samples in Florida. Belize *P. astreoides* colonies did not indicate any effect due to this extract, clustering by colony rather than treatment in nMDS analysis.

We chose *L. variegata* for this study because it commonly overgrows the edges and competes for space with the two study corals on deeper Caribbean reefs (20–25 m; [63], [64]). In addition to overgrowing corals [65], causing bleaching [15], and tissue mortality [66], previous laboratory assays demonstrated that aqueous compounds from *L. variegata* have broad-spectrum antibacterial activity against coral reef microorganisms [40]. Recent evidence also shows that brown macroalgae can produce hydrophilic sugar compounds such as dulcitol that are active against bacterial quorum sensing [67]. Dulcitol is present in *L. variegata* polar extracts based on bioassay-guided fractionation and may be responsible for some of the bacterial mediation seen during this study [40], Dobretsov, Gunasekera & Paul *unpubl. results*). *L. variegata* also produces polyphenolic compounds that not only have antibacterial activity, but also function as herbivore deterrents and digestive inhibitors, making them less likely to be consumed and more likely to proliferate on coral reefs (reviewed in [68], [69]). Furthermore, the polycyclic macrolide, lobophorolide, was identified in whole tissue extracts of *L. variegata* [70]. Although lobophorolide may be cyanobacterial in origin, it is found in concentrations sufficient for fungal inhibition on the surfaces of *L. variegata* thalli [70]. Thus, *L. variegata* has significant potential to alter microorganisms associated with competing corals, particularly because much of the activity is present in the aqueous fraction, which is more readily dispersed within the surrounding water than organic extracts.

The natural concentration of compounds found in whole algal extracts were used throughout the present study, but we recognize that the concentration of compounds at or near the surface of the algal thalli that would come into direct contact with the coral SML may be quite different. Although it is likely that aqueous compounds could be released into the surrounding water regardless of where they were found in the competing algal tissues. The *L. variegata* extracts used in this study on corals in Belize and Florida were derived from macroalgae collected in St. Thomas. Future research will address site-specific differences in algal extracts and the compounds responsible for the dramatic effects of *L. variegata* on coral associated microorganisms. Further research is also needed to identify the taxonomic and functional shifts in bacterial assemblage structure and to determine whether these shifts are detrimental to the coral host. It is also important to determine the speed at which different coral holobionts return to an initial equilibrium after the competing alga or extract is removed as another measure of species resilience.

The data presented here are an important first step toward examining the impact of macroalgal compounds on coral associated microorganisms. Although we did not provide taxonomic information for the bacterial assemblages found within the coral SML in this study, we were able to compare >300 diverse bacterial samples using DGGE. A dataset of this size allowed us to illustrate larger ecological patterns among multiple coral-macroalgal species interactions. We demonstrate that some extracts of macroalgae (e.g. *L. variegata*) can have far-reaching effects on coral-bacterial communities, while others are less effective (e.g. *Dictyota* sp.). These patterns provide new perspectives on coral-algal competition and provide support for additional research into the consequences of altering the natural state of coral microbial communities. As present-day reefs continue to undergo phase-shifts to alternative dominants that may have potent biochemical defense mechanisms [10], [71], [72], we should question what effect this will have on overall reef health and physiology.
